# Broadband Anisotropy in Terahertz Metamaterial With Single-Layer Gap Ring Array

**DOI:** 10.3390/ma12142255

**Published:** 2019-07-13

**Authors:** Liangping Xia, Hong-Liang Cui, Man Zhang, Suihu Dang, Chunlei Du

**Affiliations:** 1 School of Electronic Information Engineering, Yangtze Normal University, Chongqing 408100, China; 2 Chongqing Key Laboratory of Multi-Scale Manufacturing Technology, Chongqing Institute of Green and Intelligent Technology, Chinese Academy of Sciences, Chongqing 400714, China

**Keywords:** terahertz, metamaterial, resonance, anisotropy

## Abstract

To convert the polarization of terahertz waves pumped by a femtosecond laser in a terahertz time domain system, a broadband anisotropic metamaterial is proposed. The metamaterial is constructed with a single-layer gapped metallic ring array, which supports different resonant modes in orthogonal directions. With the aid of simulations and measurements, the anisotropy of the terahertz transmission is demonstrated and discussed. The experimental results of THz transmission in the metamaterial indicate that the anisotropic band is as wide as 0.56 THz, which accords well with our theoretical prediction.

## 1. Introduction

Terahertz (THz), ranging between 0.1 and 10 THz (3.3–333.3 cm^−1^), is the electromagnetic wave whose wavelengths fall in between those of the microwave and the optical wave. It has characteristic properties, such as relatively low photon energy, so that it is free from causing ionization damage. Meanwhile, such photon energies are in tune with finger-print spectral features of many biological molecules [1,2,3]. Benefiting from these characters, broadband THz technology is widely used in substance detection [4,5,6]. At present, typical broadband terahertz detection is based on a femtosecond laser pumped time domain system (TDS) [7,8,9]. However, in a THz-TDS, the THz radiation is linearly polarized in the common configuration of the photoconductive antenna, which limits its applications, such as measurement of chiral molecule [10,11].

A waveplate based on an anisotropic material is typically used to convert the polarization of the optical wave [12,13]. However, unlike in the case of optical waves, a naturally anisotropic material with remarkable birefringence in the THz band is elusive if not non-existent. Although man-made materials by the methods of paper stacking and carbon fiber alignment are researched to enhance the anisotropy, a thick device is required to obtain a large birefringence [14,15]. In a metamaterial with subwavelength structures, it is easy to achieve anisotropy by controlling the structural distribution [16,17,18]. However, the resultant strong anisotropy based on spectral resonances in the metamaterial invariably leads to a narrow working frequency band [19,20]. To extend the working frequency band, stacking alternating layers of different metamaterials with correspondingly different resonant frequencies are reported [21,22]. However, the thickness of such a device will inevitably increase, and the fabrication process of these multilayered metamaterials will become hopelessly complicated.

In this work, a THz metamaterial based on a single-layer metallic gapped-ring array is proposed to convert the polarization of THz waves in a wideband. With carefully designed multimode resonances supported by the metamaterial, the working frequency band is extended. Compared with previously reported THz waveplate schemes based on metamaterials, the new device is not only thin, comprising a single active layer, but is also broadband. The transmission and resonant modes are analyzed with the help of simulations and experiments. The measured THz transmission in the metamaterial shows that the anisotropy is achieved between 0.275 and 0.835 THz, in agreement with our theoretically predicted outcomes.

## 2. Structures

**Figure 1:**
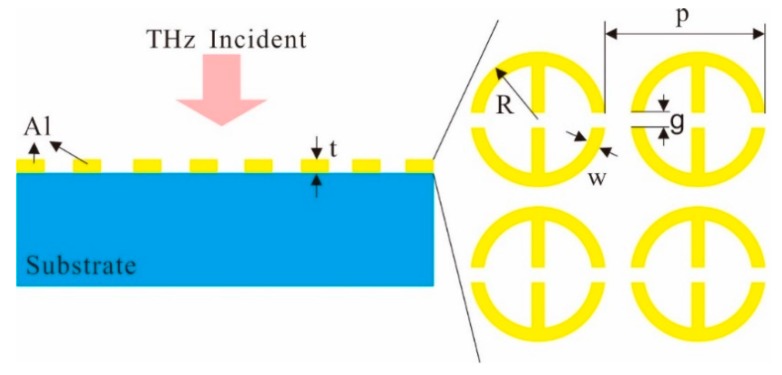
Sketch of the broadband Terahertz (THz) anisotropic metamaterial.

The configuration of the THz anisotropic metamaterial is as shown in Figure 1. The structure is composed of a single-layer metallic-ring array with period of *p* in both the *x* and *y* direction. The metallic ring with the width of *w* and outer radius of *R* is cut by the gaps with a width of *g* in the *x* direction. In the metallic ring, there are arms extended toward to the center in the *y* direction. The metallic structure is placed on the surface of a low-loss substrate. As the distributions of the metallic metamaterial are different in the orthogonal direction, it follows that the transmission properties are different in the *x* and *y* directions when the incident THz wave is on the structure, which will lead to the anisotropy in the structure.

**Figure 2:**
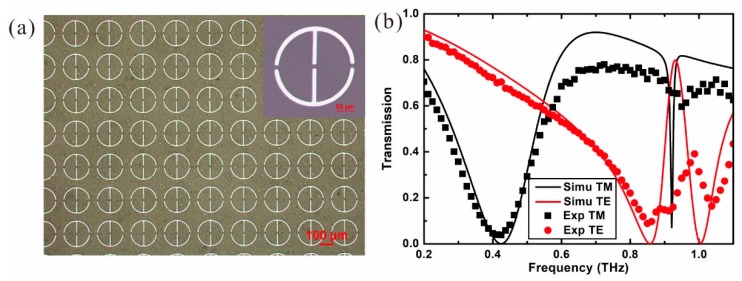
(**a**) Microscope picture of fabricated THz anisotropic metamaterial. (**b**) Simulated and measured transmission spectrum of the metamaterial in the *x* and *y* directions.

The THz anisotropy metamaterial is fabricated with the method of hard template support [23]. A polyethylene glycol terephthalate (PET) film with the thickness of 35 μm is chosen as the low-loss substrate. An aluminum (Al) film with the thickness of 100 nm is deposited onto the PET substrate by magnetron sputtering. The pattern of the metamaterial is fabricated with lithography and wet etching, resulting in a periodic array of gapped rings, as shown in Figure 2a. The microscope figure indicates that the fabricated structure is uniform and defect-free.

The THz anisotropic metamaterial is analyzed by simulations with the method of finite difference time domain (FDTD) electromagnetics. In the simulations, the parameters of the structure are the same as those of the fabrication device. The period of the ring array is *p* = 250 μm, the outer radius is *R* = 100 μm, the width *w* = 10 μm, and the gap width is *g* = 10 μm. The width of the arms in the *y* direction is the same as the width of the ring. The material parameters are ε*_PET_* = 3 and σ*_A l_* = 3.5 × 10^7^ S/m. THz wave with different polarizations and broadband frequencies is incident normally onto the metamaterial in the simulations and the obtained transmission spectra are shown in Figure 2b, with solid curves. The black curve indicates that there are two minimum points at the frequencies of *f* = 0.428 THz (Mode 1) and *f* = 0.92 THz (Mode 2) when the polarization of the THz wave is in the *x* direction (TM mode). It reveals that the metamaterial excites the resonant modes at the frequencies of the minimum points. Benefiting from the difference of the structure in the orthogonal directions, the red curve indicates that the resonant modes are changed to the frequencies of *f* = 0.857 THz (Mode 1) and *f* = 1.005 THz (Mode 2) when the polarization of the THz wave is in the *y* direction (TE mode).

The transmission of the fabricated THz anisotropic metamaterial is further measured with the THz time-domain spectroscopy system (Advanced Photonix, Inc., Ann Arbor, MI, USA), with a frequency resolution of 12.5 GHz. In Figure 2b, the black square dotted curve is for the TM mode and red circle dotted curve is for the TE mode incidence. It shows that the transmissions of the metamaterial are in excellent agreement with the FDTD simulations at the lower resonant modes. At the higher resonant modes of the TM and TE incidence, the measured results show that there is a slight blue shift of the resonant frequencies. The measured transmission of the resonant mode at *f* = 0.95 THz is not as sharp as that from the simulation, which is caused by the limitation of the frequency resolution of the THz-TDS.

## 3. Discussions

**Figure 3:**
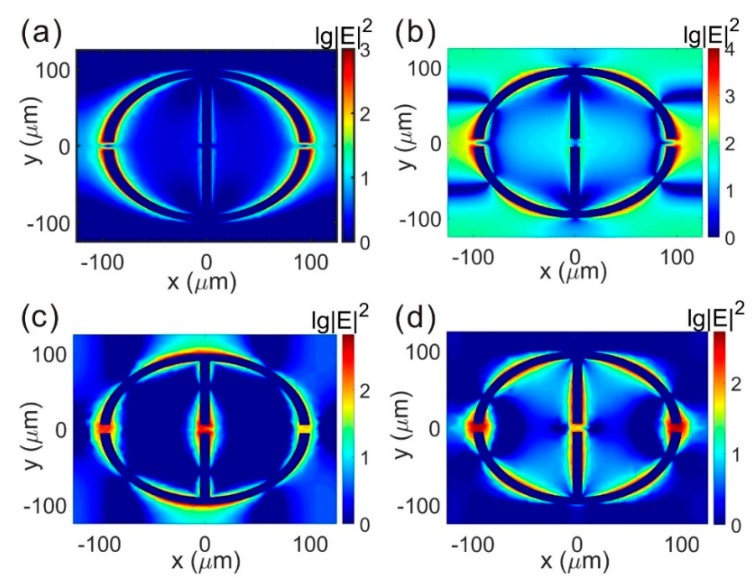
Electric field distributions of (**a**) *f* = 0.428 THz of TM incidence; (**b**) *f* = 0.92 THz of TM incidence; (**c**) *f* = 0.857 THz of TE incidence; (**d**) *f* = 1.005 THz of TE incidence.

The distributions of the electric field for the resonant modes are simulated to understand the mechanism of the resonances excited by the metallic structures. For the THz incident of TM mode, the result in Figure 3a shows that the electric field is mainly distributed around the metallic ring in the *x* direction when the resonant frequency is *f* = 0.428 THz. It also indicates that there is one resonant mode excited between the neighboring rings. When the resonant frequency is *f* = 0.92 THz, the result in Figure 3b shows that there are three resonant modes between the metallic neighboring rings. Compared with the electric field distributions of mode 1 and mode 2 for the TM incidence, the results reveal that the resonance at *f* = 0.428 THz is the first-order mode and the resonance at *f* = 0.92 THz is the third-order mode. It indicates that only the odd modes are supported under TM incidence.

For the THz incident of TE mode, the result in Figure 3c shows that, except for the electric field distribution between the metallic rings in the *y* direction, the field is also localized in the metallic gaps when the frequency is *f* = 0.857 THz. When the frequency is *f* = 1.005 THz, the electric field distribution in Figure 3d shows that the gap resonance is excited, the same as in mode 1, but the resonance between the metallic rings is changed to higher orders. Compared with the electric field distributions of mode 1 and mode 2 for the TM incidence, the results reveal that the resonance at *f* = 0.428 THz is the first-order mode and at *f* = 0.92 THz is the second-order mode. Due to the difference of the gap resonant mode and the higher ordered resonant mode between the TM and TE incidence, the property of anisotropy is obtained in the THz metamaterial.

**Figure 4:**
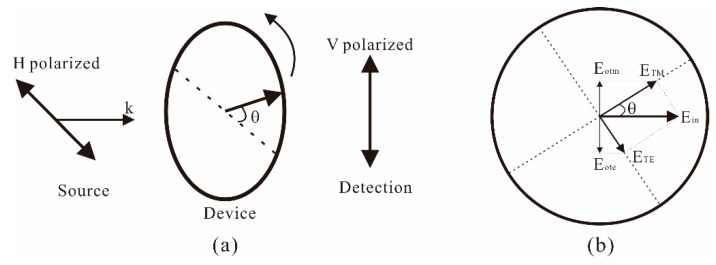
Schematic of measurement of THz anisotropy in the metamaterial.

To discuss the broadband property of the anisotropic metamaterial, the fabricated device of the THz metamaterial is measured with the setup shown in Figure 4. The polarization of the THz source is horizontal, while the THz detector detects vertically polarized radiation. In the measurement, the device of the THz metamaterial is rotated with the angle *θ*. When the device is rotated, the directions of the TM and TE incidence are also rotated; hence, the component of $E_{TE}=E_{in}\sin\;\theta$, $E_{TM}=E_{in}\cos\;\theta$. The transmitted field in the vertical direction is as follows:

(1) $$E_{o}=E_{otm}+E_{ote}$$

where $E_{otm}=E_{TM}T_{TM}$, $E_{ote}=E_{TE}T_{TE}$, $T_{TM}$ and $T_{TE}$ are the transmission coefficients of the *TM* and *TE* incidence, respectively.

**Figure 5:**
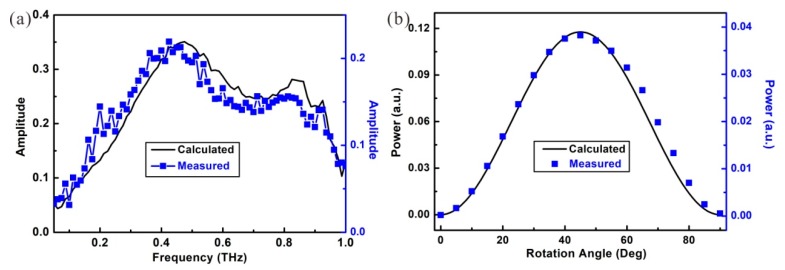
Calculated and measured THz anisotropy in the metamaterial. (**a**) Transmission amplitude vs. frequency for a rotation angle of θ = 45°. (**b**) Power of transmission vs. rotation angle at the frequency of *f* = 0.4 THz.

In the measurements, the rotation angle is fixed at θ = 45° first, and the amplitude received by the THz detector is shown as the blue square dots in Figure 5a. The result shows that the THz anisotropic transmission in the fabricated metamaterial occurs from 0.275 THz to 0.835 THz. With Formula (1), the amplitude of the transmission is also calculated and the result is shown as the black curve in Figure 5a. In the calculations, the transmission coefficients of $T_{TM}$ and $T_{TE}$ are obtained from the measured result of TM and TE transmission in Figure 2b. In comparing with the results, the curves show that the theoretical calculation agrees with the experimental result, and the 3-dB frequency band of anisotropy is as wide as 0.56 THz.

The influence of the rotation angle on the transmission is further discussed. When fixing the frequency at *f* = 0.4 THz, the measured transmission power by the THz detector is shown with the blue dots in Figure 5b. The result shows that the THz transmission in the metamaterial first increases with the rotation angle and reaches the maximum when θ = 45°. When the rotation angle increases further, the received power in the THz detector is decreased. The calculated result shown with the black curve indicates that the experimental result also agrees with the theoretical prediction. These results together demonstrate that the fabricated metamaterial could efficiently convert the polarization of the THz-TDS from linear polarization to circular polarization or elliptical polarization over a rather broad frequency range.

In addition, the performance of the THz anisotropic metamaterial was compared with the previous reported works and the results are shown in Table 1. In terms of the metallic layers and the anisotropic frequency band, although the metallic metamaterial in our device is single-layer, the frequency band is wider than the reported multi-layered devices.

## 4. Conclusions

A THz anisotropic metamaterial with a single-layer gapped metallic ring array structure was analyzed, whose resonant modes in the orthogonal direction excited by the structure were simulated. The metamaterial device was fabricated and measured, with the results of the THz transmission indicating that the anisotropic frequency band was as wide as 0.56 THz. Furthermore, the experimental results are in strong agreement with the theoretical calculations. This anisotropic metamaterial is a good candidate in polarization conversion of the typically used THz-TDS from linear polarization to quasi-circular polarization and has potential applications in fingerprint spectrum measurement of the chiral molecule. The broadband property of the metamaterial will help to cover rich fingerprint features in molecule sensing.

## Figures and Tables

**Table 1:** Performance comparison of the THz metamaterials.

THz Anisotropic Metamaterial	Metallic Layers	Frequency Band	Band Width
Split square ring [20]	Single-layer	0.58–0.7 THz	0.12 THz
Meanderline structure [20]	Double-layer	0.55–0.82 THz	0.27 THz
Grating [21]	Double-layer	1.4–1.8 THz	0.4 THz
Grating [22]	Three-layer	0.15–0.42 THz	0.27 THz
Our device	Single-layer	0.275–0.835 THz	0.56 THz
